# Supplementary material for: Cerebral blood flow and histological analysis for the accurate differentiation of infiltrating tumor and vasogenic edema in glioblastoma
Source: PLoS One. 2025 Jan 10;20(1):e0316168. doi: 10.1371/journal.pone.0316168 (PMC11723604; doi:10.1371/journal.pone.0316168)
Supplement: Attachment — Submitted filename: For_reviewer_ver5.docx [file pone.0316168.s001.docx]

**Reviewer #1:**

3. Major Concerns
   3.1. Small Sample Size: The relatively small number of patients (48 with glioblastoma and 24 with meningioma as controls) may limit the robustness and generalizability of the results. Larger studies are necessary to confirm the findings and broaden their applicability.

Answer: We thank you for your comment. We agree with your concern and have addressed it in the limitation section. This study was limited by its small sample size, which could lead to potential bias in our results. Therefore, future large-scale studies are required to validate the differentiation of infiltrating tumors and vasogenic edema with MRI perfusion. We have added the following statement to the revised manuscript (page 17, line 18): This study faces potential validation challenges. Firstly, it was limited by its small sample size, which could have led to bias in our results. Additionally, we sampled tissue stereotactically using a navigation system, which may have introduced coordinate errors during tissue sampling. Furthermore, we encountered some issues with DSC images. Reproducibility problems with DSC between patients, blurring due to the calculation method, or spatial resolution limitations may also pose difficulties. In the future, the sampling error should be validated while minimizing the effects of brain shifting and standardizing reproducible methods and analysis processes. Overall, future large-scale studies are required to address these methodological challenges and improve the ability to differentiate between NETs and Edemas on T2-weighted images.

3.2. Limited Discussion on Clinical Impact: Although the study demonstrates the utility of CBF and MTT for differentiating NETs and edemas, there is limited discussion on how these findings could translate into clinical decision-making, particularly in terms of surgical planning or modifying treatment strategies.

Answer: We thank you for your valuable comment. As you noted, this is an important point. Improving the extent of resection in the surgical treatment of glioblastoma leads to a better prognosis. Although the utility of supramarginal resection needs further validation, it is expected to contribute to improved outcomes. The T2 high-intensity lesion surrounding the contrast-enhanced lesion contains a mixture of edema and infiltrative tumor. Distinguishing between the NETs and Edemas preoperatively and resecting the infiltrative area contributes to better prognosis and preservation of neurological function. Using MRI perfusion preoperatively to differentiate between NETs and Edemas will be expected to set an accurate resection margin that will allow for an increase in the resection rate safely without worsening neurological function. Accordingly, we have added the following statement to the revised manuscript (page 16, line 21): Distinguishing between NETs and Edemas preoperatively and resecting the infiltrative area contributes to better prognosis and preservation of neurological function. Additionally, visually creating a threshold-based CBF and MTT map can enhance preoperative surgical planning. Moreover, using MRI perfusion preoperatively to differentiate between NETs and Edemas is expected to set an accurate resection margin, allowing for an increased resection rate safely without worsening neurological function.

3.3. Lack of Comparison with Other Modalities: The study would benefit from comparing DSC-MRI with other imaging techniques, such as diffusion-weighted imaging or arterial spin labeling, to better contextualize the advantages and limitations of DSC-MRI in distinguishing tumor from edema.

Answer: We thank you for your insightful comment. As you highlighted, this is an important point. We validated the differentiation of NET from Edema using DSC-MRI and ADC values. The ADC ratios were significantly higher in NETs than in Edemas (p<0.05). However, no significant difference was observed between ETs and NETs in ADC ratios. Accordingly, we have added the following statement to the revised manuscript (page 11, line 9):

No significant difference was observed in the mean CBV ratios between Edemas, NETs, and ETs (Figure 3 D). The mean CBF ratio for Edemas was significantly lower than that for NETs (p<0.01). In contrast, the mean MTT ratio for Edemas was significantly higher than that for NETs (p<0.01). However, no significant difference was observed in the CBF and MTT ratios between ETs and NETs (Figure 3 E and F). To further clarify the distinction between NETs and Edemas, we compared the validation using ADC values. The ADC ratios were significantly higher in NETs than in Edemas (p<0.05) (Figure 3 G). However, no significant difference was observed in ADC ratios between ETs and NETs.

We have added the following statement to the revised manuscript (page 16, line 8):

In addition, we validated the discrimination between NETs and Edemas using ADC values, CBF ratios, and MTT ratios. Notably, previous reports have shown that ADC values can be used as indicators of glioma proliferation [36, 37]. This background supports our investigation, as we demonstrated the potential usefulness of ADC values for discriminating between NETs and Edemas, consistent with the results of CBF and MTT ratios.

We also added the following statement to the revised manuscript (page 7, line 1):

DTI was acquired using a single-shot echo planar imaging technique with TE=80 and TR=10,000. Diffusion gradient encoding in 25 directions with b=2,000 s/mm2 and an additional measurement without the diffusion gradient (b=0 s/mm2) was performed [23].

We also added the following statement to the revised manuscript (page 7, line 12):

DTI was also acquired qualitatively apparent diffusion coefficient (ADC) values using an ADC map.

We also added the following statement to the revised manuscript (page 21, line 17): Figure 3

D, E, F, and G

Boxplots of the cerebral blood volume (CBV), cerebral blood flow (CBF), mean transit time (MTT), and apparent diffusion coefficient (ADC) ratios in non-contrast-enhancing tumors (NETs), edemas (Edemas), and enhancing tumors (ETs) based on a histological comparison using stereotactic imaging. The mean CBF ratio for Edemas was significantly lower than that for NETs (p<0.01). The mean MTT ratio for Edemas was significantly higher than that for NETs (p<0.01) The ADC ratios were significantly higher in NETs than in Edemas (p<0.05). However, no significant difference was observed in CBV ratios between NETs and Edemas.

(page 22, line 13):

Figure 5

Correlation of the ADC ratio with cell density, Ki-67, and microvessel area based on a histological comparison using stereotactic imaging. The ADC ratio shows no correlation with cell density, the Ki-67 index, or microvessel area.

4. Minor Issue
   4.1. Clarity of Data Presentation: Some tables and figures, particularly those depicting imaging data, could benefit from more detailed legends and explanations to improve interpretability (e.g., Figure 4).

Answer: We thank you for your comment. We agree with your comment. Accordingly, we have added detailed legends and explanations. We have also added the following statement to the revised manuscript (page 21, line 7):

Figure3

A, B, and C

Boxplots of the Ki-67 index, cell density, and microvessel area in non-contrast-enhancing tumors (NETs), edemas (Edemas), and enhancing tumors (ETs). The Ki-67 index, cell density, and microvessel area were significantly higher in NETs compared to Edemas (p<0.05, p<0.01, and p<0.05, respectively). The Ki-67 index and microvessel area were significantly higher in ETs than in NETs (p<0.05 and p<0.05, respectively). However, no significant difference in cell density between ETs and NETs was observed.

D, E, F, and G

Boxplots of the cerebral blood volume (CBV), cerebral blood flow (CBF), mean transit time (MTT), and apparent diffusion coefficient (ADC) ratios in non-contrast-enhancing tumors (NETs), edemas (Edemas), and enhancing tumors (ETs) based on a histological comparison using stereotactic imaging. The mean CBF ratio for Edemas was significantly lower than that for NETs (p<0.01). The mean MTT ratio for Edemas was significantly higher than that for NETs (p<0.01). The ADC ratios were significantly higher in NETs than in Edemas (p<0.05). However, no significant difference was observed in CBV ratios between NETs and Edemas.

(page 23, line 3):

Figure 7

Boxplots of the cerebral blood volume (CBV), cerebral blood flow (CBF), and mean transit time (MTT) ratios in non-contrast-enhancing tumors (NETs), edemas (Edemas), enhancing tumors (ETs) in 48 glioblastoma patients, and Edemas in the control group (Edemas-M). The mean CBF ratio for Edemas (0.56; range, 0.11–1.08) was significantly lower than that for NETs (1.64; range, 0.39–4.05) (p<0.01). The mean MTT ratio for Edemas (1.83; range, 1.22–3.30) was significantly higher than that for NETs (0.96; range, 0.34–1.60) (p<0.01). In contrast, the CBF and MTT ratios for Edemas and the controls exhibited similar tendencies.

Boxplots of the cerebral blood flow (CBF) and mean transit time (MTT) ratios in local recurrent tumors in 12 relapsed glioblastoma patients. The mean CBF ratio for the recurrent tumor was 1.136, exceeding the cut-off value of 0.943 to predict NETs. The mean MTT ratio for the recurrent tumor was 0.944, falling below the cut-off value of 1.229 to predict NETs.

(page 23, line 19):

Figure 8

The receiver operating characteristic (ROC) curve shows reliable predictions that distinguish non-contrast-enhancing tumors (NETs) and edemas (Edemas) in glioblastoma in terms of cerebral blood flow (CBF) and mean transit time (MTT). The CBF ratio (area under the curve [AUC] =0.890) effectively distinguishes between NETs and Edemas, with a sensitivity of 74.2% and a specificity of 97.1% (cut-off value =0.943, p<0.01). The MTT ratio (AUC=0.946) also effectively distinguishes between NETs and Edemas, with a sensitivity of 80.6% and a specificity of 97.1% (cut-off value =1.229, p<0.01).

4.2. ROC Analysis Application: While the ROC analysis for CBF and MTT is statistically strong, further elaboration on how these metrics might be applied in clinical settings would enhance the study's practical relevance.

Answer: We thank you for your comment. As you noted, this is an important point. Accordingly, we have added the following statement to the revised manuscript (page 13, line 5): We presented the probability map applied to NETs and Edemas lesions in two patients with glioblastoma and meningioma (Figures 9 and 10). The probability map predicting NETs indicated a CBF ratio over 0.943, represented in orange. The area colored blue corresponded to Edema CBF ratios below 0.943. Additionally, the probability map predicting NETs showed an MTT ratio below 1.229, also colored orange, while the area colored blue applied to Edema MTT ratios above 1.229. Notably, the area of the probability CBF map for Edemas containing NETs did not necessarily align with the area in the probability MTT map for Edemas in both glioblastoma and meningioma cases.

We also have added the following statement to the revised manuscript (page 17, line 8): In particular, the proposed CBF and MTT cut-off values for distinguishing NETs in two patients with glioblastoma and meningioma exhibited potential for differentiating Edemas from infiltrating tumors, albeit with limitations. For instance, the Edema probability CBF map showed the presence of NETs, while the probability MTT map did not identify them, even in the case of meningioma. This suggests that the MTT map was better at detecting edema than the CBF map. Ultimately, a more effective analysis is warranted to determine the accuracy of these cut-off values using 11C-methionine (MET)-PET, which allows for more accurate delineation of tumor extension than anatomical imaging achieved with MRI[40].

We also have added the following statement to the revised manuscript (page 24, line 5):

Figure 9

Illustrative case of glioblastoma. It shows probability maps for cerebral blood flow (CBF) and mean transit time (MTT), which predict non-contrast-enhancing tumors (NET) and Edema, fused with T2-weighted images. The probability CBF map predicts a NET where the CBF ratio is higher than 0.943, and it predicts Edema where the CBF ratio is lower than 0.943. The probability MTT map predicts a NET where the MTT ratio is lower than 1.229, and it predicts Edema where the MTT ratio is higher than 1.229.

Figure 10

Illustrative case of meningioma. It shows probability maps for cerebral blood flow (CBF) and mean transit time (MTT), which predict non-contrast-enhancing tumors (NETs) and Edemas, fused with T2-weighted images. The probability CBF map predicts a NET where the CBF ratio is higher than 0.943, and it predicts Edema where the CBF ratio is lower than 0.943. The probability MTT map predicts NET where the MTT ratio is lower than 1.229, and it predicts Edema where the MTT ratio is higher than 1.229.

4.3. Control Group Explanation: The inclusion of meningioma patients as controls for vasogenic edema is valuable; however, a more thorough explanation of their selection and relevance to glioblastoma would strengthen the rationale for using this group as a comparison.

Answer: We thank you for your insightful feedback. Accordingly, we have added the flowchart for the case accumulation of glioblastoma and meningioma as controls. We have also added Figure 1 to outline the criteria used in this study. We have added the following statement to the revised manuscript (page 20, line 5):

Figure 1

The process of patient selection for inclusion in the study. We retrospectively collected the medical records of 75 patients diagnosed with *IDH*-wildtype glioblastoma between January 2017 and January 2023 at our institution. Of those 75 patients, 27 were excluded from the study due to tumors with unsatisfactory images and/or prior radiotherapy before MRI examination, leaving 48 eligible for radiological analysis. In 14 of these cases, stereotactic sampling was performed. During the review period, local recurrent analysis was also conducted. Of the 48 patients, 36 were excluded from the recurrent analysis due to recurrent tumors with unsatisfactory images, biopsy or partial resection, lack of recurrence, or no follow-up. Additionally, we retrospectively collected the medical records of 58 patients diagnosed with meningioma between January 2017 and January 2023 at our institution. Of those 58 patients, 38 were excluded from the study due to tumors with unsatisfactory images, Grade 2/3 meningioma, or infratentorial tumors.

5. Suggestions for Improvement
   5.1. Larger Sample Sizes in Future Studies: Expanding the cohort size in future studies would improve the statistical power and help validate the findings across a broader population of glioblastoma patients.

Answer: We thank you for your comment. We agree with your suggestion. This study was limited by its small sample size. This is an exploratory study, and whether this study’s results are universal remains unclear. Future large-scale studies are required to validate the differentiation of infiltrating tumors and vasogenic edema with MRI perfusion. Accordingly, we have added the following statement to the revised manuscript (page 18, line 5): There were limitations to this study due to the small sample size and the exploratory nature of the study. Additionally, the low number of specimens may lead to bias in our results, potentially hindering sound statistical analyses. It also remains unclear whether our findings are useful for supratotal resection. Further analysis of the vascular structure and permeability involved in tumor infiltration is necessary to differentiate NETs from Edemas in terms of tumor infiltration mechanisms. Furthermore, large-scale studies would enhance statistical power and validate the proposed differentiation between NETs and Edemas on T2-weighted images from a prognostic perspective.

5.2. Incorporation of Prognostic Data: Adding data on clinical outcomes, such as patient survival and progression-free intervals, would provide a more comprehensive understanding of the clinical significance of distinguishing NETs from edema using DSC-MRI.

Answer: We thank you for your valuable comment. As you noted, this is an important point. We evaluated preoperative CBF and MTT ratios in 12 local recurrent cases at recurrent lesions. The mean CBF and MTT ratios were 1.136 and 0.944, respectively. Cut-off values to predict NETs for CBF and MTT ratios were 0.943 and 1.229, respectively. CBF ratios at recurrent lesions were mainly plotted over cut-off values to predict NETs. Similarly, MTT ratios at recurrent lesions were mainly plotted under cut-off values to predict NETs. This suggests that CBF and MTT ratios can also predict recurrence sites. Accordingly, we have added the following statement to the revised manuscript (page 13, line 15):

**Prognostic values of MR perfusion imaging parameters**

We evaluated the 12 local recurrent cases for preoperative CBF ratios and MTT ratios at recurrent lesions. All lesions were applicable in NETs and turned into enhancing tumors at recurrence. The preoperative mean CBF ratio for the recurrent lesion was 1.136, above the cut-off value of 0.943 to predict NETs. The preoperative mean MTT ratio for the recurrent lesion was 0.944, below the cut-off value of 1.229 to predict NETs (Figures 7 and 8).

We have also added the following statement to the revised manuscript (page 17, line 4): Furthermore, analysis based on local recurrent cases has revealed the potential prognostic value of DSC-MRI, as DSC-MRI parameters can effectively discriminate between recurrent tumors and predict future tumor recurrence [38, 39]. Building on this, our study suggests that the CBF and MTT ratios may potentially predict recurrent lesions. In particular, the proposed CBF and MTT cut-off values for distinguishing NETs in two patients with glioblastoma and meningioma exhibited the potential for differentiating Edemas from infiltrating tumors, albeit with limitations. For instance, the Edema probability CBF map showed the presence of NETs, while the probability MTT map did not identify them, even in the case of meningioma. This suggests that the MTT map was better at detecting edema than the CBF map. Ultimately, a more effective analysis is warranted to determine the accuracy of these cut-off values using ^11^C-methionine (MET)-PET, which allows for more accurate delineation of tumor extension than anatomical imaging achieved with MRI[40].

Comparison with Other Imaging Techniques: Including a comparison with alternative imaging modalities could highlight the specific advantages of DSC-MRI and provide a more balanced view of its role in differentiating NETs from edema.

Answer: We thank you for your comment. We agree with your suggestions. As we responded to **Reviewer #1** 3.3, we compared the validation using ADC values to enhance clarity in distinguishing between tumors and edema using DSC-MRI. The differentiation between NET and edema using ADC values yielded results similar to those obtained with CBF and MTT ratios. Since ADC values are known to be associated with glioma proliferation, the validity of differentiation using CBF and MTT ratios was demonstrated.

6. Conclusions and Recommendations
   The manuscript offers valuable insights into the use of DSC-MRI for distinguishing NETs from vasogenic edemas in glioblastoma. The integration of imaging data with histopathological analysis is a significant strength of the study, and the findings have the potential to impact clinical management. However, the small sample size and limited discussion on clinical applications slightly reduce the study’s overall impact. Expanding the sample size and incorporating additional outcome data would further enhance the value of the research.

Answer: We thank you for your comment and agree with your suggestions. This study was limited by its small sample size. This is an exploratory study, and whether this study’s results are universal remains unclear. As we intend to further enhance the value of this study, we analyzed alternative imaging modalities and additional outcome data. We have added the comparison with ADC values, which present the proliferation in glioma. We have also added the prognostic values to prove the clinical significance of distinguishing NETs from Edema using DSC-MRI.

We have added the following statement to the revised manuscript (page 10, line 19):

**Correlation of MR perfusion imaging parameters and ADC values with Ki-67 index, cell density, and microvessel area values based on a histological comparison using stereotactic imaging**

We have added the following statement to the revised manuscript (page 11, line 14):

To further clarify the distinction between NETs and Edemas, we compared the validation using ADC values. The ADC ratios were significantly higher in NETs than in Edemas (p<0.05) (Figure 3 G). However, no significant difference was observed in ADC ratios between ETs and NETs.

We have added the following statement to the revised manuscript (page 13, line 15):

**Prognostic values of MR perfusion imaging parameters**

We evaluated the 12 local recurrent cases for preoperative CBF ratios and MTT ratios at recurrent lesions. All lesions were applicable in NETs and turned into enhancing tumors at recurrence. The mean CBF ratio for the recurrent tumors was 1.136, above the cut-off value of 0.943 to predict NETs. The mean MTT ratio for the recurrent tumors was 0.944, below the cut-off value of 1.229 to predict NETs (Figures 7 and 8).

We have also added the following statement to the revised manuscript (page 17, line 4): Furthermore, analysis based on local recurrent cases has revealed the potential prognostic value of DSC-MRI, as DSC-MRI parameters can effectively discriminate between recurrent tumors and predict future tumor recurrence [38, 39]. Building on this, our study suggests that the CBF and MTT ratios may potentially predict recurrent lesions. In particular, the proposed CBF and MTT cut-off values for distinguishing NETs in two patients with glioblastoma and meningioma exhibited the potential for differentiating Edemas from infiltrating tumors, albeit with limitations. For instance, the Edema probability CBF map showed the presence of NETs, while the probability MTT map did not identify them, even in the case of meningioma. This suggests that the MTT map was better at detecting edema than the CBF map. Ultimately, a more effective analysis is warranted to determine the accuracy of these cut-off values using 11C-methionine (MET)-PET, which allows for more accurate delineation of tumor extension than anatomical imaging achieved with MRI[40].

**Reviewer #2:**

1. Quantitative and qualitative research for data type approach in statistical results and analysis is important. Especially, Figure 1 is the conception of this study in data analysis. Unfortunately, The reviewer didn't see any tables describing it in sufficient detail.

Answer: We thank you for your comment and agree with your concern. Accordingly, we have added Table 1, which presents the sampling data.

2. The biggest issue is that the data analysis should more robust and so, the authors are going to have to find a way to properly demonstrate their data. How about the power analysis in statistics?

Answer: We thank you for your insightful comment. This is an exploratory study without a predefined threshold; therefore, it is impossible to calculate the power. While the analysis you presented appears meaningful, we regret that we cannot conduct it, considering the exploratory nature of this study.

3. The image mark-up plays an important role in results. The Figure 1 is divided into three colors marked segments. What is your groundwork based on these color-marked ?

Answer: We thank you for your insightful comment. Accordingly, we have changed the color for ET, NET, and Edema. ET, NET, and Edema were marked in red, orange, and blue, respectively.

4. Ki-67 labeling index is an important indicator of tumor cell proliferation in glioma, which can only be obtained by postoperative biopsy regarding microvascular proliferation as a crucial histological feature of glioblastoma.Could you show the different ROIs correspondingly in histological features in this study? Additionally, please discuss this point in the Discussion section.

Answer: We thank you for your suggestion. As you noted, this is an important point. Accordingly, we have added the following statement to the revised manuscript (page 12, line 4): We visually represented the interrelationships between cell density, Ki-67 index, and microvascular area in stereotactically evaluated ETs, NETs, and Edemas using a 3D scatter plot (Figure 6). The plots for Edemas were distributed over a smaller area for cell density, Ki-67 index, and microvascular area compared to those for NETs and ETs. Additionally, while the plots for NETs and ETs overlapped, the plots for ETs were located in areas with higher values for cell density, Ki-67 index, and microvascular area than those for NETs.

We have added the following statement to the revised manuscript (page 14, line 16):

Moreover, histological evaluation using a 3D scatter plot showed that the spatial distribution of NETs was different from that of Edemas.

We have added the following statement to the revised manuscript (page 22, line 18): Figure 6

3D scatter plot representing the interrelationships between cell density, the Ki-67 index, and microvascular area in stereotactically evaluated ET, NETs, and Edemas. The plots for Edemas were distributed in a smaller area for cell density, the Ki-67 index, and microvascular area compared to those for NETs and ETs. Although the plots for NETs and ETs were mixed, the plots for ETs were found in areas with higher values for cell density, the Ki-67 index, and microvascular area compared to the plots for NETs.

5. Data Implement
   5.1. Regarding the edema, T2*-based dynamic susceptibility contrast (DSC) MRI is a good point for approach, however, correlation coefficient value interpretation is weak for low correlation. Could you discuss this part in the discussion?

Answer: We thank you for your insightful comment. The correlations among the CBF ratio, MTT ratio, Ki-67 index, cell density, and microvessel area presented weak coefficient values. This could be due to several reasons. We sampled tissue stereotactically using a navigation system. This might have induced co-ordinate errors during tissue sampling. Furthermore, we also have some potential problems with DSC images. Reproducibility issues with DSC between patients, blurring due to calculation methods, or spatial resolution limitations may lead to potential problems. We need to verify the sampling error by minimizing the effect of brain sifting, standardizing reproducible methods, and refining analysis processes. Future large-scale studies are required to validate the methodological problems.

We have added the following statement to the revised manuscript (page 17, line 18):

This study faces potential validation challenges. Firstly, it was limited by its small sample size, which could have led to bias in our results. Additionally, we sampled tissue stereotactically using a navigation system, which may have introduced coordinate errors during tissue sampling. Furthermore, we encountered some issues with DSC images. Reproducibility problems with DSC between patients, blurring due to the calculation method, or spatial resolution limitations may also pose difficulties. In the future, the sampling error should be validated while minimizing the effects of brain shifting and standardizing reproducible methods and analysis processes. Overall, future large-scale studies are required to address these methodological challenges and improve the ability to differentiate between NETs and Edemas on T2-weighted images.

5.2. What else about the study limitations except a small sample size? Please add more in Discussion.

Answer: We thank you for your insightful comment.

Accordingly, we have added the following statement to the revised manuscript (page 17, line 18): This study faces potential validation challenges. Firstly, it was limited by its small sample size, which could have led to bias in our results. Additionally, we sampled tissue stereotactically using a navigation system, which may have introduced coordinate errors during tissue sampling. Furthermore, we encountered some issues with DSC images. Reproducibility problems with DSC between patients, blurring due to the calculation method, or spatial resolution limitations may also pose difficulties. In the future, the sampling error should be validated while minimizing the effects of brain shifting and standardizing reproducible methods and analysis processes. Overall, future large-scale studies are required to address these methodological challenges and improve the ability to differentiate between NETs and Edemas on T2-weighted images.

**Reviewer #3:**

1. General Comments: However, a major point of concern arises from the lack of correlation between CBV and these same parameters, despite the fact that several studies in the literature suggest that CBV is typically more sensitive than CBF in reflecting neovascularization, especially in gliomas.

Answer: We thank you for your comment. As you noted, this is an important point. We have discussed this in Question 2: Explanation for the Discrepancy between CBF and CBV.

2. Explanation for the Discrepancy between CBF and CBV: It would be beneficial for the authors to provide a more in-depth explanation or hypothesis as to why CBV did not show a significant correlation with microvessel area, cell density, or Ki-67 index, while CBF did. Given that both CBF and CBV are considered reliable markers of neovascularization, the observed discrepancy raises important questions. In some studies, CBV has been shown to better capture neovascularization than CBF. The authors should address whether the lack of correlation could be due to technical factors, such as the selection of arterial input function (AIF) or the specific post-processing algorithms used in the study, particularly since CBV calculation is known to be more sensitive to such variables.

Answer: We thank you for your valuable comment and agree with your concern. The images in our study were post-processed with a dedicated software package. The previous reports that CBV reflected neovascularization in glioblastoma were also postprocessed using a dedicated software package. However, the software was different from ours. The different software used for image processing of CBV measurements and the different algorithms may have influenced the results.

Accordingly, we have added the following statement to the revised manuscript (page 15, line 20):

In our study, CBV ratios in ETs, NETs, and Edemas were not significantly different. We particularly focused on the accurate placement of the ROI for ETs in the solid part of the enhancing tumor area to exclude the confounding effects of tumor heterogeneity, signal loss due to cyst formation, hemorrhage, and large blood vessels. Therefore, the CBV ratio of the ET may not always represent the maximum values. On the other hand, previous reports have determined CBV to be a useful estimation tool for angiogenesis in glioblastoma [34, 35]. These reports measured CBV using post-processing with a dedicated software package. However, it is possible that the different software used for image processing of CBV measurements and the varying algorithms may have influenced the results that differ from ours. The methods used for ROI design and imaging processes for CBV may have led to an increased CBF ratio without a significant increase in the CBV ratio in ETs.

3. The Role of Vessel Co-option: Additionally, the authors may wish to consider the role of vessel co-option, which is prominent in glioblastomas. Vessel co-option could explain why CBF increases without a corresponding rise in CBV, as pre-existing vasculature is co-opted by infiltrating tumor cells. This phenomenon may result in increased blood flow (CBF) without a significant increase in vessel volume (CBV), particularly in the NET regions. A discussion on whether this mechanism could account for the differential behavior of CBF and CBV would provide valuable context to the findings.

Answer: We thank you for your insightful comment. When assessing the tumor lesions, we particularly focused on the accurate placement of the ROI in the solid part of the enhancing tumor area to exclude the confounding effects of tumor heterogeneity, signal loss due to cyst formation, hemorrhage, and large blood vessels. Therefore, the rCBV of the ET may not always represent the maximum values. The method used for ROI design can result in increased CBF without a significant increase in CBV in the ETs.

We have added the following statement to the revised manuscript (page 15, line 20):

In our study, CBV ratios in ETs, NETs, and Edemas were not significantly different. We particularly focused on the accurate placement of the ROI for ETs in the solid part of the enhancing tumor area to exclude the confounding effects of tumor heterogeneity, signal loss due to cyst formation, hemorrhage, and large blood vessels. Therefore, the CBV ratio of the ET may not always represent the maximum values. On the other hand, previous reports have determined CBV to be a useful estimation tool for angiogenesis in glioblastoma [34, 35]. These reports measured CBV using post-processing with a dedicated software package. However, it is possible that the different software used for image processing of CBV measurements and the varying algorithms may have influenced the results that differ from ours. The methods used for ROI design and imaging processes for CBV may have led to an increased CBF ratio without a significant increase in the CBV ratio in ETs.

We have also added the following statement to the revised manuscript to clarify the ROI setting for the measurement of parameters. (page 8, line 7): The ROI of identical size was also set manually in contralateral normal area for each ROI for Edema, NET, and ET.

4. Potential Influence of Tumor Stage or Region: It may also be worthwhile to discuss whether the stage of tumor progression or the specific tumor region examined could influence the sensitivity of CBF versus CBV in detecting neovascularization. Early-stage tumors or regions with active infiltration might show different perfusion characteristics than more established tumor regions. Clarifying whether the study accounted for such factors would strengthen the interpretation of the results.

Answer: We thank you for your insightful comment. This study did not consider the stage of tumor progression or the specific tumor region. To clarify the size of the tumors studied, we have added the volume of the contrast-enhanced lesions to Table 1.

5. Presentation of Representative Images: To further enhance the clarity and impact of the study, it would be valuable to include representative images from both glioblastoma and meningioma cases. Specifically, providing clear examples of DSC-MR perfusion images with corresponding CBF, CBV, and MTT maps for typical cases of NETs and Edemas in glioblastoma, as well as for vasogenic edema in meningioma, would help readers better understand the radiological differences described in the study. These images would also visually support the conclusions drawn from the quantitative data and improve the overall comprehensibility of the findings.

Answer: We thank you for your valuable comment. As you noted, this is an important point. We have presented a case with a colored map using CBF and MTT ratios (Figures 9 and 10), according to Reviewer 1 4.2.

6. Inclusion of Color Scale Units and LUT Bars in Figure 1: A minor but important detail for improving the clarity of Figure 1 is the addition of the color scale units and LUT (Lookup Table) bars for the CBV, CBF, and MTT maps. Including these will allow readers to interpret the quantitative perfusion values more effectively. Clear color scales and units are essential for accurately assessing the radiological findings presented and ensuring that the visual data aligns with the statistical analysis discussed in the manuscript.

Answer: We thank you for your insightful comment. Accordingly, we have added the Color Scale Units and LUT Bars to Figure 2.

Other changes

Figure numbers have been changed to reflect the revised content.
